# Association between metabolic syndrome, cognitive dysfunctions, and peripheral inflammation in schizophrenia

**DOI:** 10.1192/j.eurpsy.2024.513

**Published:** 2024-08-27

**Authors:** A. Kancsev, O. Kelemen, S. Kéri

**Affiliations:** ^1^Department of Psychiatry and Psychotherapy, SZSZBMK Jósa András University Teaching Hospital, Nyíregyháza; ^2^Department of Behavioral Sciences, Albert Szent-Györgyi Medical School, University of Szeged, Szeged; ^3^Department of Psychiatry, Bács-Kiskun County Hospital, Kecskemét; ^4^Department of Cognitive Science, Budapest University of Technology and Economics; ^5^National Institute of Mental Health, Neurology, and Neurosurgery, Budapest; ^6^Department of Physiology, Albert Szent-Györgyi Medical School, University of Szeged, Szeged, Hungary

## Abstract

**Introduction:**

Metabolic syndrome (MetS) is of primary clinical interest because of its harmful impact on the general health and quality of life of patients with psychotic disorders. Paradoxically, MetS is associated with impaired cognitive functions in patients receiving antipsychotics primarily shown to improve cognition (e.g., clozapine and olanzapine).

**Objectives:**

In this study, we aimed to investigate the relationship between MetS, cognitive functions, and peripheral inflammation.

**Methods:**

The participants were 154 patients with schizophrenia. Fifty-seven patients met the criteria of MetS. We evaluated cognitive functions with the Repeated Battery for the Assessment of Neuropsychological Status (RBANS). The Positive and Negative Syndrome Scale (PANSS) quantified the clinical symptoms. We also measured the plasma levels of IL-6 and C-reactive protein (CRP). In addition to conventional statistics, we also calculated Cohen’s effect size (d) and Bayes Factors (BF10).

**Results:**

Results revealed that patients with MetS exhibited worse cognitive function relative to patients without Mets in attention (d = 0.19, BF10 = 2.3) and delayed memory (d = 0.25, BF10 = 5.7). No significant between-group differences existed in immediate memory, visuospatial functions, and language. The MetS and non-MetS groups did not differ in positive, negative, or general symptoms. Higher IL-6 levels were associated with worse delayed memory (r=-0.56, BF10=34.6).

**Conclusions:**

Our results suggest that MetS-associated cognitive dysfunctions are less severe than reported in the literature: it was confined to two cognitive domains, the effect size was small, and the Bayesian evidence level was weak. Peripheral inflammation may mediate the association between MetS and long-term memory dysfunctions.

**Disclosure of Interest:**

None Declared

